# Account of Diseases in an Eastern District of London, from March 20, to April 20, 1803

**Published:** 1803-05-01

**Authors:** 


### *Account of Diseases in an Eastern District of London, from March 20, to April 20, 1803.*

| | | | |
|---|---|---|---|
| ACUTE DISEASES. | | Gastrodynia | 5 |
| Synocha Catarrhalis | 39 | Dyspepsia | 7 |
| Rheumatismus Acutus | 5 | Amenorrhœa | 12 |
| Peripneumonia Notha | 8 | Menorrhagia | 4 |
| CHRONIC DISEASES. | | Hysteria | 3 |
| Dyspnœa | 10 | Epilepsia | 1 |
| Phthisis Pulmonalis | 4 | Hypochondriasis | 4 |
| Hæmoptoe | 3 | Dysuria | 3 |
| Hydrops Pectoris | 2 | Herpes | 4 |

Lepra

| | |
|---|---|
| Lepra - - - - - - - 1 | INFANTILE DISEASES. |
| Rheumatismus Chronicus 23 | Aphthæ - - - - - - 3 |
| PUERPERAL DISEASES. | Febris Mesenterica - - 4 |
| Menorrhagia Lochialis - 5 | Tinea - - - - - - - 2 |
| Enuresis - - - - - - 2 | Pertussis - - - - - - 3 |
| Dolores post Partum - - 6 | |

The disease which has been considered as the reigning epidemic, and which was pretty largely described in our last report, has not yet entirely disappeared. It has, however, abated in its violence, and the number of patients afflicted by it is considerably smaller. To a disease, the consequences of which have been so general, the attention of the Faculty must have been particularly directed; and we accordingly find, that an account of its nature and symptoms, as well as of the most successful mode of treatment, has been given by different practitioners in a distinct publication, notice of which has been taken in the Numbers of this Journal.

As some steps are now taking by a society which enjoys the advantage of an extensive correspondence, with a view to obtain the most compleat history of this disease; it may be expected that a very general view of it will be afforded, not only as it has prevailed in the metropolis and its environs, but also in different parts of this kingdom, and in some more remote parts of the globe.

